# STM-induced ring closure of vinylheptafulvene molecular dipole switches on Au(111)†

**DOI:** 10.1039/d2na00038e

**Published:** 2022-09-20

**Authors:** Kwan Ho Au-Yeung, Tim Kühne, Oumaima Aiboudi, Suchetana Sarkar, Olga Guskova, Dmitry A. Ryndyk, Thomas Heine, Franziska Lissel, Francesca Moresco

**Affiliations:** Center for Advancing Electronics Dresden, TU Dresden 01062 Dresden Germany francesca.moresco@tu-dresden.de; Leibniz Institute of Polymer Research 01069 Dresden Germany; Faculty of Chemistry and Food Chemistry, TU Dresden 01062 Dresden Germany; Institute for Materials Science, TU Dresden 01062 Dresden Germany; Theoretical Chemistry, TU Dresden 01062 Dresden Germany

## Abstract

Dihydroazulene/vinylheptafulvene pairs are known as molecular dipole switches that undergo a ring-opening/-closure reaction by UV irradiation or thermal excitation. Herein, we show that the ring-closure reaction of a single vinylheptafulvene adsorbed on the Au(111) surface can be induced by voltage pulses from the tip of a scanning tunneling microscope. This cyclization is accompanied by the elimination of HCN, as confirmed by simulations. When inducing lateral movements by applying voltage pulses with the STM tip, we observe that the response of the single molecules changes with the ring closing reaction. This behaviour is discussed by comparing the dipole moment and the charge distribution of the open and closed forms on the surface.

## Abstract 2

Ring-closure reaction of a single vinylheptafulvene adsorbed on the Au(111) surface induced by voltage pulses from the tip of a scanning tunneling microscope.

## Graphical abstract

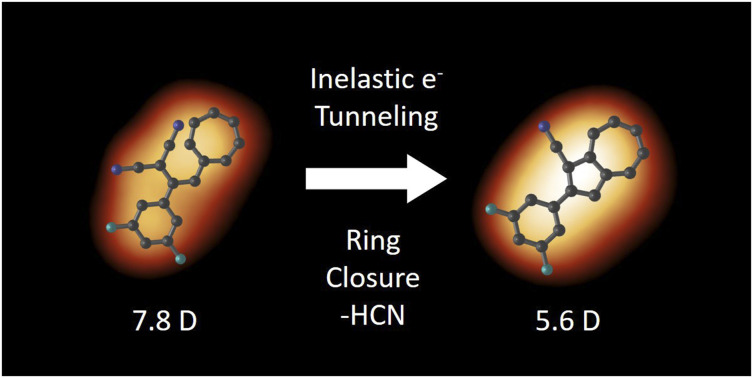

## Introduction

Controlling chemical reactions at the single molecule level represents a fascinating scientific goal, which can be now reached for model compounds under the tip of a scanning tunneling microscope (STM). The scanning tip of the STM not only allows the imaging of reaction precursors and products, but furthermore can be used as an extremely precise local tool to induce a reaction: positioning the metal tip on a molecule makes it possible to investigate chemical conversions by inelastic tunneling electrons and by locally applied electric fields.^1^ A very recent example is the selective multiple isomerization reactions that were obtained by tuning the voltage pulses from the tip and imaged by atomic force microscopy (AFM).^2^

Beside non-reversible chemical reactions, like controlled dissociations or bond formation, reversible conformational changes, rotations, and molecular switching can be induced by the STM tip, practically converting energy from inelastic electron tunneling into movement,^3^ thereby storing the energy for a finite time. On the search for new approaches for energy storage, such phenomena are presently in the focus of research. Molecular switches are in this respect particularly interesting and a few examples of optically switchable molecule investigated for energy storage have been recently reported.^4^

Chemistry on surface follows own rules. The absolute absence of solvents and the confinement to a 2D geometry make predictions based on solution chemistry very difficult.^5^ It is therefore crucial to correctly assign precursors and reaction products. When the compounds adsorb flat on the supporting surface and do not diffuse under the action of the tip during the imaging time, the assignment can be done by high-resolved STM or AFM imaging with functionalized tip.^2,6^ In most cases, however, the molecules are non-planar and/or mobile so that STM or AFM images are not able to resolve the single atoms or bonds, and a correct assignment of the chemical structure requires comparison with simulated images based on density functional theory (DFT).^7,8^

**Scheme 1:**
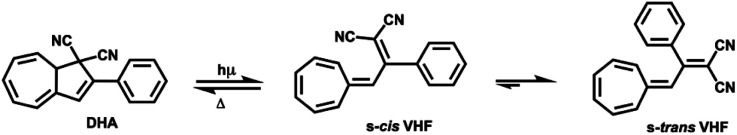
Parent DHA structure conversion to *s-cis*-VHF and *s-trans*-VHF.

Molecular switches are a class of molecules, which can be toggled between two stable forms *via* an external stimulus, *e.g.* optical, thermal stimuli or electric fields.^9–11^ Photoisomerization may result in the *cis*–*trans* isomerization of a substituted double bond (*e.g.* azobenzene and diarylethylene^12,13^), or in a more complex rearrangement of chemical bonds, such as ring-closure and ring opening. Several examples of photochromic switches undergoing ring-opening and closing are described in the literature, for instances, spiropyrans and dihydroazulene/*s-cis*-vinylheptafulvene (DHA/*s-cis*-VHF) pairs.^14,15^ In case of the latter, the initial product of the photo-induced retroelectrocyclization at 367 nm is *s-cis*-VHF, which can either undergo a ring closure back to DHA, or convert into the more stable *s-trans*-conformer (Scheme 1).

The back-reaction to the DHA isomer is induced thermally.^16^ The conversion from *s-cis*-VHF to its conformer *s-trans*-VHF takes place in solution, while in solid state the isomerization is inhibited.^17^

The conversion of DHA to the corresponding *s-cis*-VHF is associated with significant changes of the chemical and physical properties.^18,19^ In the gas phase, the *s-cis*-VHF structure is planar, it has a larger dipole moment of 7.8 D, and significantly different UV-vis absorption maxima as indicated by the experimentally observed color change from yellow (DHA) to dark red (*s-cis*-VHF). Meanwhile, the closed ring of the DHA form has a lower dipole moment (5.6 D). At the single molecule level, the conversion of DHA/*s-cis*-VHF pairs has been investigated by the mechanically controllable break junction technique.^20^

Molecular switches can be studied by STM after adsorption on a surface,^21–26^ inducing conformational changes not only by the conventional photo-stimulus, but also using the tip of the STM by mechanical manipulation,^27^ electric fields,^24,25^ or inelastic tunneling electrons.^21^

Herein, we investigated by low-temperature STM the on-surface cyclization in molecules based on DHA/*s-cis*-VHF to understand and locally control electronic properties and dipole moment. We synthetized DHA-F_2_ molecules and sublimated them on the Au(111) surface. Upon adsorption, we observed the nearly complete transformation of DHA-F_2_ into *s-cis*-VHF-F_2_. We induced with the STM tip a cyclization reaction accompanied by the elimination of HCN on single *s-cis*-VHF-F_2_. The variation of dipole moment and charge distribution between the two forms is reflected by the different mobility under the STM tip. The assignment of precursors and products relies on the comparison with density functional theory (DFT) calculations and STM image simulations.

## Results and discussion

### Rational design and synthesis

**Scheme 2:**
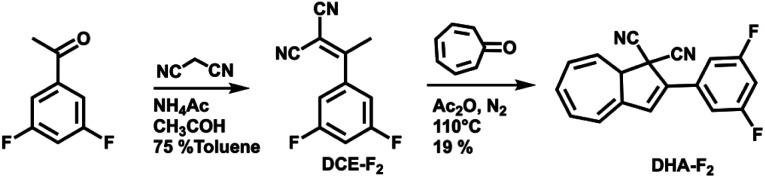
Synthesis route for the preparation of DHA-F_2_.

For this study, the ideal photo (chromic) switch is characterized by two spectrally well-separated states that are stable, chemically inert, and differ as much as possible regarding the dipole moment. Based on the results of the calculated dipole moments in the gas phase (see Table S1 in the ESI†), we targeted the derivative DHA-F_2_ (see Scheme 2) carrying fluoro substituents in the 3 and 5 positions: the dipole moment is expected to change strongly when switching from the DHA to the *s-cis*-VHF form (difference of more than 2.8 D), leading to the expectation that the two isomers will respond distinctively different to the external stimuli of the STM tip. The new 3,5-difluoro substituted dihydroazulene DHA-F_2_ was prepared in two steps starting from commercially available 3′,5′-difluoroacetophenone in an overall yield of 15% based on a procedure described by Nielsen and co-workers.^28^ First, a Knoevenagel reaction between the 3′,5′-difluoroacetophenone and malononitrile gave dicyanoethene derivative DCE-F_2_ in a good yield (75%). The subsequent formation of DHA-F_2_ was achieved by condensation of tropone and DCE-F_2_ in the presence of acetic anhydride at 110 °C. DHA-F_2_, a yellow solid, was obtained with 19% yield.

### Adsorption on Au(111)

**Fig. 1:**
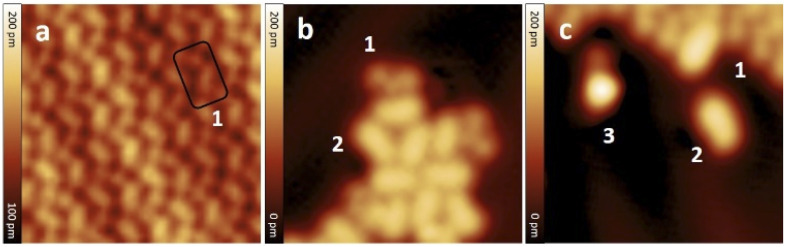
STM images of molecules adsorbed on Au(111) after sublimation: (a) detail of a molecular island formed by identical molecules 1, (b) small assembly of molecules 2 with a few 1, (c) detail showing the three different molecules denoted 1, 2, and 3. Molecules 1 are elbow-shaped, 2 appear as a peanut shape, and 3 have a clearly distinct higher protrusion at one end. STM images size: 8 nm × 8 nm; *V* = 0.5 V and *I* = 7.7 pA.

After the synthesis, DHA-F_2_ was introduced in a glass crucible into the UHV chamber and sublimated at temperatures in the range 95 °C to 110 °C on the Au(111) surface kept at room temperature. After cooling the sample to 5 K, STM images (see Fig. S13 and S14,† and a detail in Fig. 1a) show large islands formed by identical elbow-shaped molecules we denoted 1. Furthermore, a minority of peanut-shaped molecules denoted 2 (Fig. 1b and c) are visible, either forming small islands or assemblies that mostly cover edge locations of the Au(111) herringbone reconstruction. Finally, a very few high-protrusion molecules denoted 3 could be identified (Fig. 1c). By counting the three species on a typical large STM image (40 nm × 40 nm) we observe about 96% of 1, about 4% of 2, and only less than 1% of 3 (in Fig. S14†). Please note that it is difficult to distinguish the few molecules 3 from defects or impurities, due to their nearly spherical shape.

**Fig. 2:**
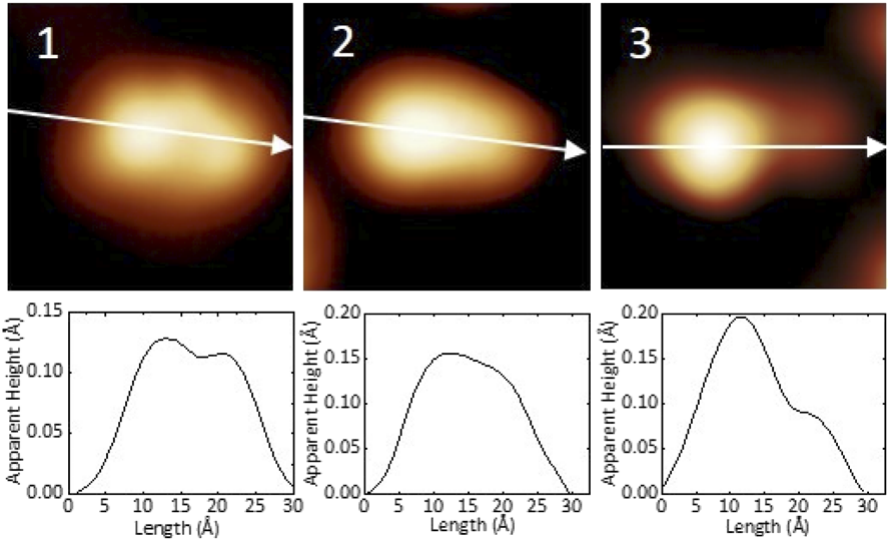
STM images and corresponding linescans of the three adsorbed species 1, 2 and 3 upon adsorption. The linescans have been taken along the white arrows. STM images size: 3 nm × 3 nm, *V* = 0.5 V and *I* = 7.7 pA.

In Fig. 2 we present detailed STM images of the three observed species with the corresponding linescans across the axis of the single molecules. Please note that to improve the resolution, molecule 1 was separated from an island edge by lateral manipulation, similarly to the case described later in Fig. 4.

As one can see, molecule 1 features two lobes at both ends and a smaller off-center part in-between. Molecule 2 appears as a less-structured peanut shape, while molecule 3 shows a distinct and higher protrusion at one end. High-resolved CO-terminated tip STM images (as recorded for example with the same setup for long acenes synthetized on-surface^6^) are not possible in the present case because the molecules are not adsorbed flat on Au(111) and are mobile during scanning. An attempt for molecule 2, which is the less mobile, is reported in Fig. S15.† As evident from Fig. 1 and 2, the identities of the three molecules cannot be determined solely relying on STM topographies. Hence, we went a step further investigating the molecules adsorbed on the Au(111) surface by DFT calculations and simulated STM images.

### Identification of the adsorbed molecules

Quantum chemistry calculations based on DFT permits to reach quantitative agreement with STM experiments allowing the identification of substances on the atomic scale, and the reaction pathways in on-surface reactions.^7^ The simulation of STM images can be further improved considering a realistic atomistic system including the STM tip and the substrate, both connected to semi-infinite electrodes and calculating the current with the Green function technique.^29^ Such method was used in the present case, having for example already demonstrated to successfully support structure assignment after on-surface chemical reactions,^30^ dissociation reactions and formation of metalorganic nanostructures.^31^

**Fig. 3:**
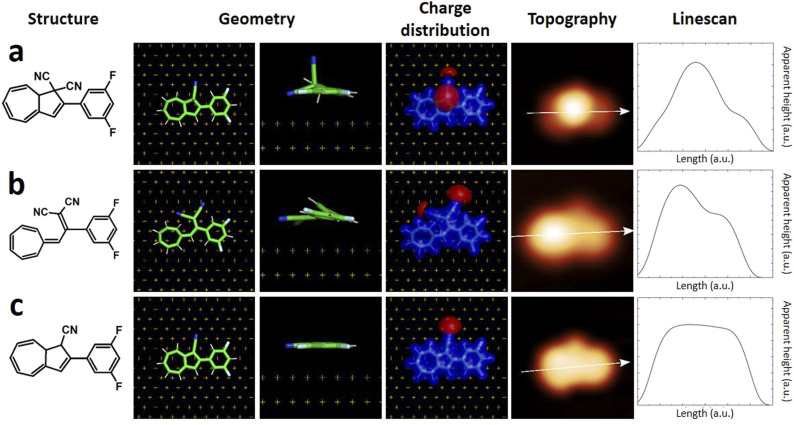
(left to right) Chemical structure, calculated adsorption geometry on Au(111), charge distribution, simulated STM image and linescans of (a) DHA-F_2_, (b) *s-cis* VHF-F_2_, (c) monocyano-DHA-F_2_.

We first calculated the adsorption geometries of the two species we expected to find on the surface, *i.e.* DHA-F_2_ (Fig. 3a) and *s-cis*-VHF-F_2_ (Fig. 3b). For each molecule, Fig. 3 shows the molecular structure, the calculated adsorption geometry on Au(111), the simulated STM image, and the corresponding simulated linescans along the molecule.

**Scheme 3:**
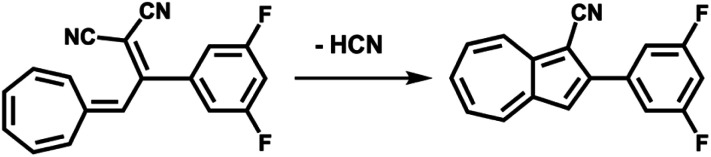
Conversion from *s-cis*-VHF-F_2_ (1) to the closed ring form monocyano-DHA-F_2_ (2) taking place upon adsorption on Au(111) and by inelastic tunneling electrons under the STM tip.

By comparing experimental STM images and linescans (Fig. 2) with simulated ones (Fig. 3) we observe that:

(a) DHA-F_2_ is adsorbed on Au(111) with the second CN group pointing perpendicular away from the surface. The corresponding simulated STM topography presents therefore a high single lobe. This geometry is not compatible neither with species 1 nor with 2, but in very good agreement with the few observed molecules 3.

(b) The most common molecule 1 forming an elbow-shaped structure well matches to the simulation for *s-cis*-VHF-F_2_ in Fig. 3b, where both CN groups are relatively flat on the surface giving rise to an elbow form in the simulated STM topography. We conclude therefore that most of the DHA-F_2_ molecules reacted to *s-cis*-VHF-F_2_ during adsorption on the surface because it is the more stable isomer, or because of exposition to day light.

(c) Considering molecule 2, we observed a shape similar to 3 but with a missing lobe, suggesting the removal of HCN upon adsorption (Scheme 3). The elimination of HCN has been described before,^19^ and monocyano derivatives were found after prolonged cycling of DHA/*s-cis*-VHF pairs in molecular solar thermal energy storage (MOST) devices.^32^ We simulated therefore the adsorbed geometry and STM images of monocyano-DHA-F_2_ closed-ring form (Fig. 3c). As one can see, the simulated STM image is in excellent agreement with the observed form 2 (Fig. 2), confirming the assignment, which is also compatible with the high resolved CO-tip image of 2 reported in Fig. S15.†

On the basis of chemical considerations and the excellent agreement between theory and experiment, we therefore assign species 1 to *s-cis*-VHF-F_2_, 2 to monocyano-DHA-F_2_, and 3 to DHA-F_2_.

### STM tip-induced on-surface reaction

**Fig. 4:**
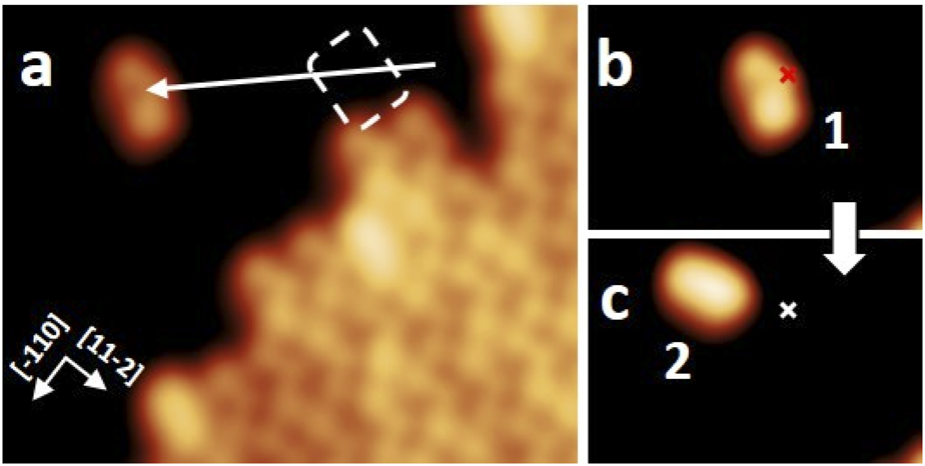
STM induced reaction on a single molecule 1. (a) STM image of an ordered molecular assembly. Lateral manipulation is applied (*V* = 10 mV, *I* = 8 pA), where the white arrow indicates the trajectory of the STM tip. (b and c) On-surface reaction event of the molecule 1 isolated in (a). (b) A constant height voltage pulse (*V* = 2.5 V, *I* ≈ 1.4 nA; marked position) is applied above the molecule 1 that was isolated in (a). (c) A lateral displacement (about 1.5 nm) was induced, and the appearance of the molecule has changed to molecule 2. The red and white marks indicate the tip position of the voltage pulse before and after the reaction. All STM images with the sizes of (a) 10 nm × 8 nm and (b and c) 6 nm × 4 nm were obtained under the conditions of *V* = 0.5 V and *I* = 7.7 pA.

To study the proposed reactions on single molecules, we separated molecule 1 (*s-cis*-VHF-F_2_) from the edge of the island since isolated 1 is not present on the gold surface. This process of isolation has been achieved by STM lateral manipulation in constant current mode^27,33,34^ (Fig. 4a). In this particular sequence of manipulations (complete sequence in Fig. S18†), the molecule 1 has shown a lateral displacement starting from the dotted position, then following the trajectory of the tip to a free adsorption position on the Au(111) terrace (white arrow; Fig. 4a).

To trigger the ring-closure reaction on the surface, individual voltage pulses were applied on molecule 1, the process is described in detail in the Methods section. Fig. 4b shows a STM topography before applying a voltage pulse above molecule 1. The red cross indicates the position of the tip during the voltage pulse. Upon applying a 2.5 V voltage pulse, a sudden current drop can be recorded (see Fig. S17† for a plot of typical current signal over time). Based on the subsequent STM topography (Fig. 2c), we can identify a conversion of 1 to 2 (together with a small displacement) induced by inelastic tunneling electrons, as shown in Scheme 3. This behavior was reproducibly observed for all induced reactions, typically with voltage pulses ranging from 2.3 V to 2.5 V. All attempts on transformation from 2 to 1 were not successful even with higher voltage bias parameters (typically with voltage pulse > 3.0 V the probability rises to cause molecular decomposition or partial cleavage of molecules).

Altogether, we have demonstrated that the open-form *s-cis*-VHF-F_2_1 can be locally transformed to the closed-ring monocyano-DHA-F_2_2 by voltage pulses with the STM tip on the Au(111) surface. Molecule 2 has never underwent a ring-opening reaction, possibly due to the adsorption on the gold surface,^25^ or to the loss of HCN. No voltage pulses were applied to molecule 3, which is very rare (less than 1%) and not clearly distinguishable from impurities and defects.

The tip-induced conversion from molecule 1 to 2 (Scheme 3) is confirmed by calculations, suggesting that the reaction can be driven by the electric field existing between tip and metal surface during the voltage pulses. The calculations show that a positive homogeneous electric field of 1 V Å^−1^ (applied to the substrate respect to the tip) is able to pull the molecule apart and may break the C–C bond inducing the HCN elimination and the cyclization reaction (Fig. S20†).

### Voltage pulse induced movement and the role of dipole moment

Voltage pulses and inelastic tunneling electrons can induce, apart from chemical reactions, lateral movement of single molecules on the surface.^31,35–38^ Hence, we investigated the movement of 1 and 2 by applying STM voltage pulses. The constant height voltage pulses were applied with the STM tip to both forms 1 and 2 after having positioned the tip laterally close to a single molecule (see also Methods for details). Note that a lower voltage (typically *V* < ±1.8 V) compared to the parameters used for inducing ring closure was chosen in order to avoid any chemical reactions but sufficient to induce movement across the surface.

**Fig. 5:**
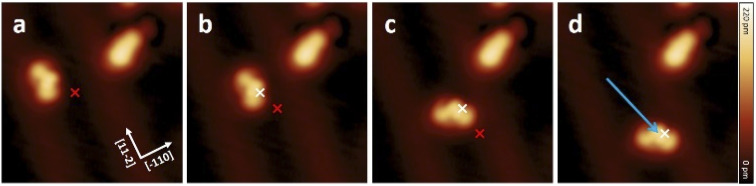
(a–d) Sequence of STM topographic images showing the movement of a molecule 1 induced by constant height STM voltage pulses (*V* = −1.8 V, *I* ≈ 50 pA) on Au(111). The red and white marks indicate the position of the tip during the voltage pulses before and after each displacement, respectively. The molecule follows the direction of induced pulses from centre to centre to a displacement of about 3.2 nm. All STM images (8 nm × 8 nm) were obtained under the conditions of *V* = 0.5 V and *I* = 7.7 pA.

Fig. 5 shows a movement sequence of a molecule 1 after applying individual voltage pulses (*V* = −1.8 V and *I* ≈ 50 pA) with the tip at the marked positions.

The red and white marks indicate the position of the tip during the voltage pulse before and after each displacement, respectively. During the pulses (where the typical pulsing time window is 10 s), a sudden increase in the absolute tunneling current revealed the movement of the molecule (see Fig. S19† for the typical current signal over time). After the subsequent imaging with low tunneling current (*I* ≈ 8 pA), the center of the molecule is at the position of the voltage pulse, indicating an attractive interaction to the local pulse. In this particular manipulation sequence, three excitation steps result in the molecule travelling 3.2 nm across the surface. Further manipulations have shown that such controlled movement can be induced regardless to the bias polarity. Interestingly, after converting molecule 1 into 2 by a voltage pulse, the movement behavior has been changed significantly, and 2 cannot be moved with similar pulsing parameters (see also Fig. S18†).

Since the movement behavior has drastically changed after the reaction from 1 to 2, it is worth to explore the physical properties of the two different forms adsorbed on the gold surface. Based on the calculated results, *s-cis*-VHF-F_2_ (1) has a higher net dipole moment of 8.75 D, and monocyano-DHA-F_2_ (2) and DHA-F_2_ (3) share the same net dipole moment of 5.55 D calculated from the on-surface geometry (more details in Table S2†). On the other hand, from the calculated charge distribution (Fig. 3), the negative charge tends to be concentrated in the electron-rich CN moieties. By comparing the charge distribution of 1 and 2 in Fig. 3, we can observe that a higher negative charge concentration on the CN side of 1 leads to the higher dipole moment, where the different movement behavior has a very good agreement with such a dipole moment difference. One can argue that dipole moment is a decisive factor for inducing movement on the surface by the STM tip.^38^ However, we have recently shown^31^ that the charge distribution of molecules can provide more specific details than the dipole moment, suggesting that charge separation and higher multipole moments can play a crucial role in the voltage-pulse induced movement of molecules on surface.

## Conclusions

In this article, we studied the adsorption, switching events, and the dipole moment of DHA-F_2_/*s-cis*-VHF-F_2_ molecules by STM manipulation on the Au(111) surface, transforming *s-cis*-VHF-F_2_ into monocyano-DHA-F_2_. By applying STM voltage pulses, we have shown that *s-cis*-VHF-F_2_ undergoes an on-surface cyclization reaction and is converted into monocyano-DHA-F_2_ by the elimination of HCN. The calculated charge distribution suggests that the tip-induced movement corresponds to the strengths of the charge separation, and thus the dipole moment difference between *s-cis*-VHF-F_2_ and monocyano-DHA-F_2_.

As an outlook, we notice that different substituent groups could significantly influence the reaction time and stability of the product, and also the adsorption of the metal surface can influence the opening and closing ring reaction. Further experiments are planned with different substituents, which can also contribute to decoupling the molecule from the metal surface.

## Methods

DHA-F_2_ molecules were evaporated at 95 °C for 15 s on a Au(111) surface kept at room temperature (25 °C). Before evaporation, the samples were cleaned by subsequent cycles of Ar^+^ sputtering and annealing to 450 °C. STM experiments were performed using a custom-built instrument operating at a low temperature of *T* = 5 K under ultrahigh vacuum (*p* ≈ 1 × 10^−10^ mbar). All shown STM images were recorded in constant-current mode with the bias voltage applied to the sample.

In order to induce chemical reactions, voltage pulse experiments were performed by positioning the STM tip at a fixed height above a molecule (non-contact) with the feedback loop switched off and applying a voltage *V*_m_ within a definite time window (from *t* = 1 s to 180 s in our cases) to the molecule per sample. The tip height was set with reference to the constant current stable tip position so that the current reached the intended value necessary to trigger the required action. Before and after applying a voltage pulse, STM images taken at low bias voltage (*V* = 500 mV) were recorded.

All lateral manipulations were performed in constant-current mode. The lateral manipulation procedure involves three steps: (1) allowing the tip to vertically approach the molecules under a small bias and current to increase the tip–molecule interaction, (2) laterally driving the tip parallel to the surface in a precisely controlled trajectory, and (3) retracting the tip to normal scanning position. The STM captures images before and after each manipulation.

For geometry optimization and charge distribution calculations, we used the DFT method as implemented in the CP2K software package (https://cp2k.org) with the Quickstep module.^39^ We applied the Perdew–Burke–Ernzerhof exchange-correlation functional,^40^ the Goedecker–Teter–Hutter pseudo-potentials^41^ and the valence double-ζ basis sets, in combination with the DFT-D2 method of Grimme^42^ for van der Waals correction. The calculations of STM topography images were performed by the DFTB+XT code from TraNaS OpenSuite (https://tranas.org/opensuite), partially based on the DFTB+^43,44^ software package. We also used the density functional based tight-binding method with auorg-1-1 parametrization^45,46^ as implemented in the DFTB+ package. We considered a realistic atomistic system including the STM tip and the substrate, both connected to semi-infinite electrodes. The simulation of STM images in the constant-current mode was done based on the current calculations by the Green function technique.^29^ The data is analysed, and the images are made by the PyMOL Molecular Graphics System, Version 2.4 open-source build, Schrödinger, LLC. Calculated lengths and apparent heights may differ between theory and experiment due to the ideal atomic tip used in calculations. We therefore label calculated apparent heights and lateral dimensions as arbitrary units.

## Author contributions

F. M. and F. L.: conceptualization; K. H., A.-Y., T. K., S. S. and F. M.: STM investigations; O. A. and F. L.: chemical synthesis and chemical investigations; O. G.; D. A. R. and T. H.: theory and calculations. All authors contributed to discussion and writing. All authors have given approval to the final version of the manuscript.

## Conflicts of interest

There are no conflicts to declare.

## Supplementary Material

NA-004-D2NA00038E-s001
