# Supplementary material for: STM-induced ring closure of vinylheptafulvene molecular dipole switches on Au(111)
Source: Nanoscale Adv. 2022 Sep 20;4(20):4351–7. doi: 10.1039/d2na00038e (PMC9552919; doi:10.1039/d2na00038e)
Supplement: NA-004-D2NA00038E-s001 [file NA-004-D2NA00038E-s001.pdf]

Electronic Supplementary Material (ESI) for Nanoscale Advances.
This journal is © The Royal Society of Chemistry 2022

STM-induced ring closure of vinylheptafulvene molecular dipole switches on Au(111)

Supporting Information

Kwan Ho Au-Yeung[1], Tim Kühne[1], Oumaima Aiboudi[2], Suchetana Sarkar[1], Olga Guskova[2], Dmitry A. Ryndyk[3,4], Thomas Heine[4], Franziska Lissel[2]*, Francesca Moresco[1]*

[1] Center for Advancing Electronics Dresden, TU Dresden, 01062 Dresden, Germany

[2] Leibniz Institute of Polymer Research, 01069 Dresden, Germany, and Faculty of Chemistry and Food Chemistry, TU Dresden, 01062 Dresden, Germany

[3] Institute for Materials Science, TU Dresden, 01062 Dresden, Germany

[4] Theoretical Chemistry, TU Dresden, 01062 Dresden, Germany

* Email: francesca.moresco@tu-dresden.de; lissel@ipfdd.de

Table of Contents

General information 3

Synthesis of 2-(3,5-difluorophenyl)azulene-1,1(8a*H*)-dicarbonitrile 4

NMR Spectroscopy 6

Thermal back reaction of VHF-F_2 monitored by ^{1}H NMR 9

IR spectroscopy 10

TGA-FTIR Data 11

UV-Vis Spectra and switching studies 12

Switching Studies of DHA-F_2/VHF-F_2 12

Calculated dipole moment in gas phase 13

Calculated dipole moment of the observed molecules 13

Additional overview STM image 14

Isolation of single molecule 1 15

Additional data of switching molecule 1 to 2 16

Tip-induced movement sequence before and after conversion 16

Additional data of moving molecule 1 17

Calculations with applied electric field 18

General information

All the starting materials were purchased from commercial suppliers (e.g. abcr GmbH, Alfa Aeser and Sigma Aldrich) and were used without any prior purification. All reactions and manipulations of air or moisture sensitive materials were performed with standard vacuum-line and Schlenk techniques under nitrogen. All reactions and manipulations involving light sensitive compounds (e.g. DHA) were conducted in a dimmed lab with yellow light foil protection on the fume hood. Column chromatography was performed on 40–63 μm SiO_2, and thin layer chromatography (TLC) was performed on commercially available precoated silica plates with fluorescence indicator and examined under UV light. With regards to the DHA compounds, a color change from yellow to red upon exposure to UV light indicates a conversion to VHF. The pure DHA was stored in glassware shielded from light by wrapping with aluminum foil.

NMR-spectra of ^{1}H and ^{13}C nuclei were recorded on a Bruker Avence III 500MHz (^{1}H 500 MHz, ^{13}C 125 MHz) spectrometer. The residual proton signal of the deuterated solvents was used as an internal standard. The NMR signal of DMSO was assigned to $\delta = 2.50$ ppm (^{1}H) and $\delta = 39.53$ ppm (^{13}C), of chloroform ($CDCl_3$) to $\delta = 7.26$ (1H) and $\delta = 77.16$ ppm (^{13}C) and of benzene (C_6D_6) to $\delta = 7.16$ (^{1}H) and $\delta = 128.06$ (^{13}C) ppm. IR spectra were obtained using an evacuated Bruker Vertex 80v FTIR spectrometer, working at a resolution of 4 cm^{-1} for 100 scans in the wavelength range of 4000 – 600cm^{-1}. The UV/Vis absorption spectroscopy measurements were carried out using a 1 cm-path-length cuvette on a Perkin Elmer "Lamda800" instrument. UV/Vis absorption spectra were obtained by scanning the wavelengths from 700 to 200 nm. QA thermogravimetric analyzer (TG) from TA Instruments, High Res TGAQ 5000, was used for thermogravimetric analysis. Thermal decomposition experiments were carried out under nitrogen purge. Degradation products were identified by matching FTIR spectra with those of the reference library.

Synthesis of 2-(3,5-difluorophenyl)azulene-1,1(8a*H*)-dicarbonitrile

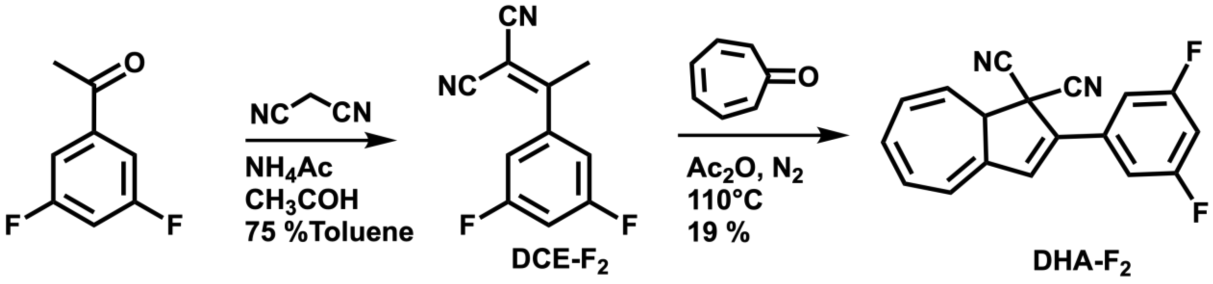

Scheme S1: Synthesis of DHA-F_2

Synthesis of DCE-F_2

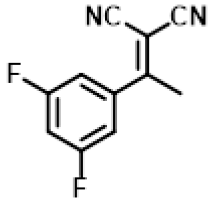

2-[1-(3,5-difluorophenyl)ethylidene]malanonitrile

To a stirring solution of 3',5'-difluoroacetophenone (2.0 g, 12.4 mmol) in toluene (100 mL) were added malanonitrile (2.74 g, 41.6 mmol) and NH_4OAc (3.84 g, 25.0 mmol) dissolved in acetic acid (10 mL). The reaction mixture was heated at 170°C for 2h. The reaction was cooled to room temperature and diluted with Et_2O (50 mL), washed with water (2 x 100 mL) and brine (100 mL). The organic phase was dried with $MgSO_4$ and the solvent removed on a rotary evaporator. The crude product was purified via column chromatography on silica gel using a mixture of n-hexane/DCM 5:1 as eluent. DCE-F_2 (1.94 g, 75%) was obtained as a white powder in a good yield. **^{1}H NMR** (500 MHz, $CDCl_3$): δ 2.61 (s, 3H), 6.98 (dt, 1H), 7.05 (dd, 2H) ppm. **^{13}C NMR** (125 MHz, $CDCl_3$): δ172.37, 164.15, 162.14, 138.5, 111.80, 110.54, 107.19, 87.00, 24.28 ppm.

Synthesis of DHA-F_2

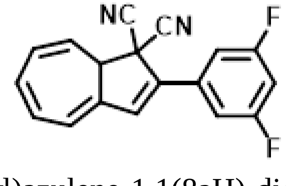

2-(3,5-difluorophenyl)azulene-1,1(8aH)-dicarbonitrile (DHA-F_2)

In a 50 mL two-neck round bottom flask equipped with a magnetic stirrer, 2-[1-(3,5-difluorophenyl)ethylidene]malanonitrile DCE-F_2 (0.53 g, 2.6 mmol) was dissolved in acetic anhydride (10 mL). Tropone (0.25 g, 2. 36 mmol) was added to the solution and the reaction mixture was heated to reflux (110°C) and stirred in the dark under N_2 atmosphere overnight. After cooling to room temperature, the reaction mixture was diluted with toluene (50 mL) to

assist *in vacuo* acetic anhydride evaporation. The resulting residue was purified by column chromatography using toluene as eluent. The column was covered with alumina foil to exclude light. Purification gave the pure product DHA-F_2 (DHA-Ph-F_2) as a crystalline yellow solid (0.135 g, 19%).

^{1}H NMR (500 MHz, DMSO): δ 3.98 (s, 1H), 5.72 (dd, 1H), 6.35 (m, 1H), 6.51 (m, 2H), 6.60 (m, 1H), 7.40 (dt, 1H), 7.48 (dd, 2H), 7.64 (s, 1H) ppm. **^{13}C NMR** (125 MHz, DMSO): δ163.83, 161.75, 137.76, 137.32, 135.88, 133.72, 131.65, 130.75, 127.65, 119.97, 114.72, 112.73, 109.09, 105.28, 50.50, 45.10 ppm. **IR** (FTIR, cm^{-1}): 3087, 2875, 2247 ($\nu_{C\equiv N}$) 1605, 1594, 1534, 1475, 1349, 1185, 992, 931, 874, 846, 699 cm^{-1}.

Photochemical Ring Opening of DHA-F_2

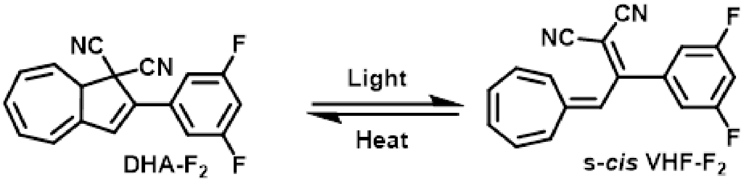

A solution of 100 mg of DHA-F_2 in 100 mL chloroform was subjected to UV irradiation (365 nm) (500 µW/cm^2) for 2h at 25°C. Within minutes, the color of the solution changes from yellow to red, indicating the conversion of DHA-F_2 to VHF-F_2. The solvent is removed at 40°C under continuous irradiation and the remaining residue is immediately chromatographed on silica gel using a mixture of petroleum ether/DCM 1:1 as eluent. After the first pale yellow fraction of unreacted/reconverted DHA-F_2 has been collected, the eluent is changed to pure DCM, and a more slowly migrating, intensive red band is eluted under UV exposure. The solvent was removed under continuous irradiation while keeping the temperature at a maximum of 30°C, finally affording s-*cis* VHF-F_2 as deep red solid (78% yield).

^{1}H NMR (500 MHz, C_6D_6): δ 6.34-6.30 (m, 3H), 5.92 (s, 1H), 5.71 (d, 1H), 5.59-5.52 (m, 2H), 5.45 (t, 1H), 5.33 (dd, 1H), 5.14-5.10 (m, 1H) ppm. **^{13}C NMR** (125 MHz, C_6D_6): δ163.83, 162.13, 152.73, 141.86, 137.51, 137.32, 135.05, 134.73, 131.15, 129.89, 122.13, 119.39, 118.28, 114.62, 114.10, 109.05, 105.57, 104.86, 78.53 ppm. **IR** (FTIR, cm^{-1}): 3071, 2923, 2856, 2206 ($\nu_{C\equiv N}$) 1620, 1504, 1482, 1442, 1349, 1252, 1209, 1185, 1120 cm^{-1}.

NMR Spectroscopy

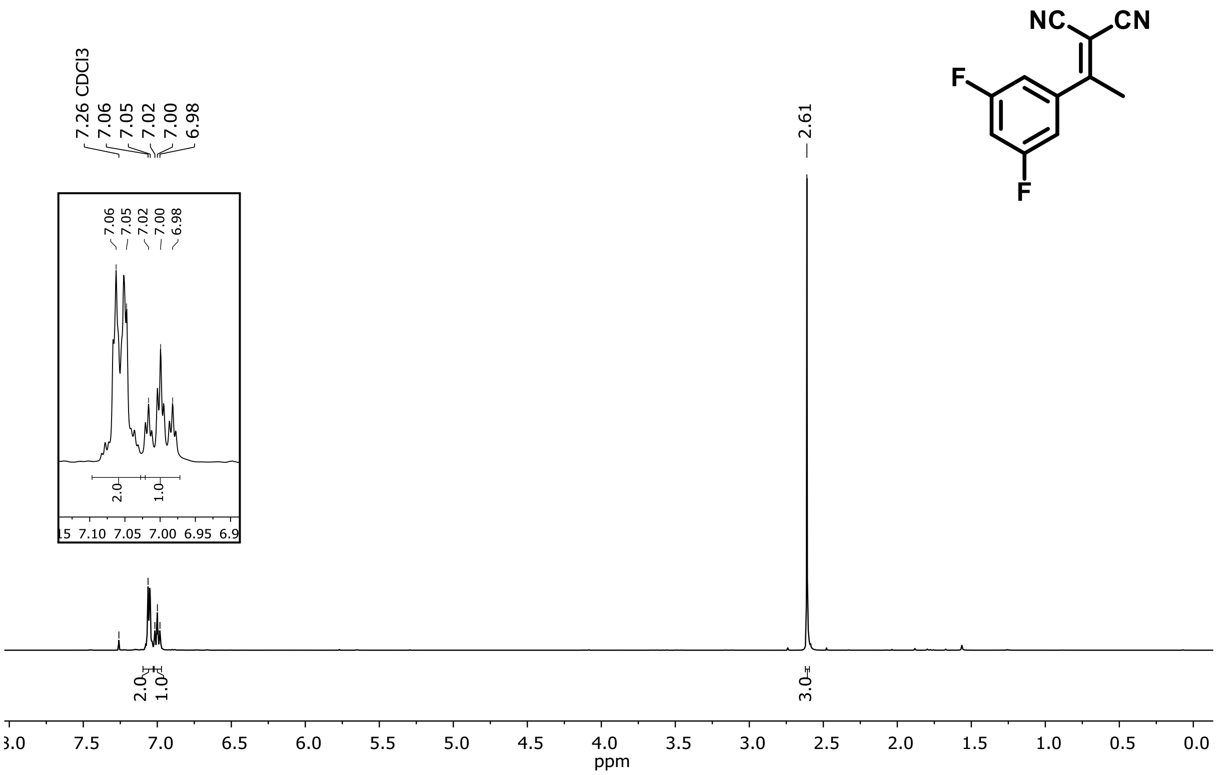

Figure S1. ^{1}H NMR of DCE-F_2 in $CDCl_3$

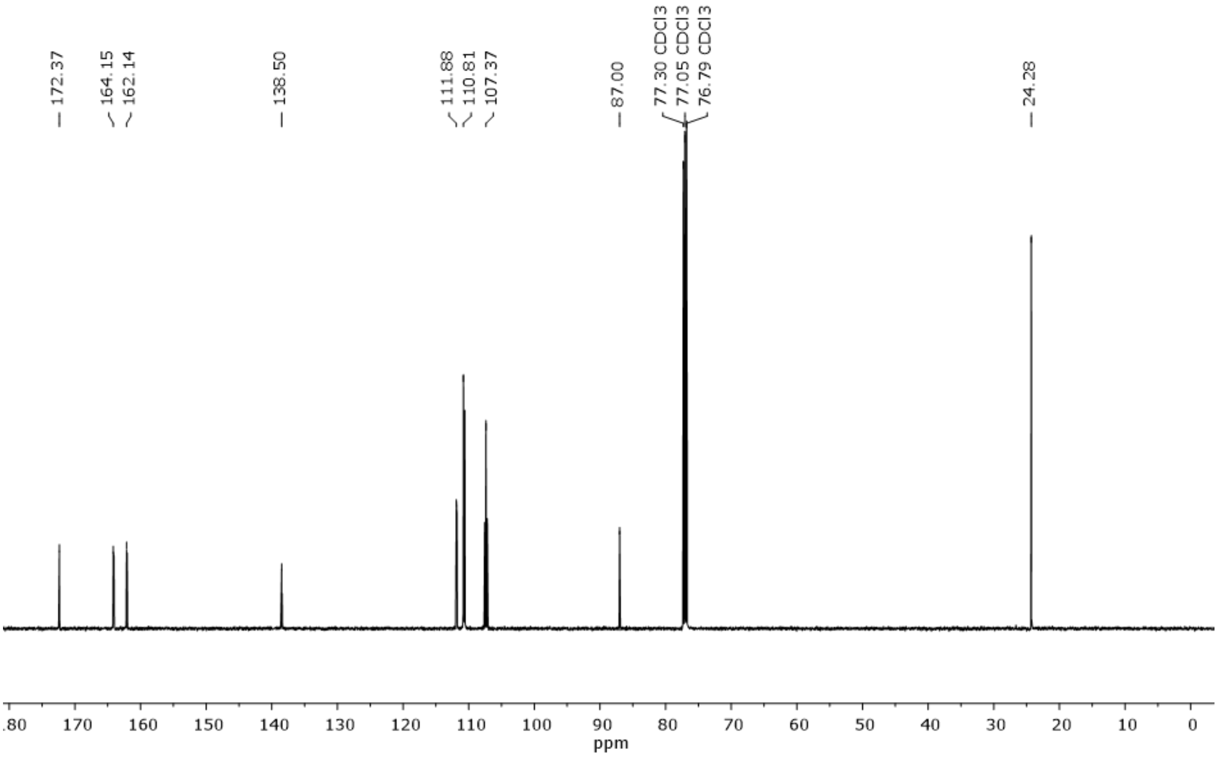

Figure S2. ^{13}C NMR of DCE-F_2 in $CDCl_3$

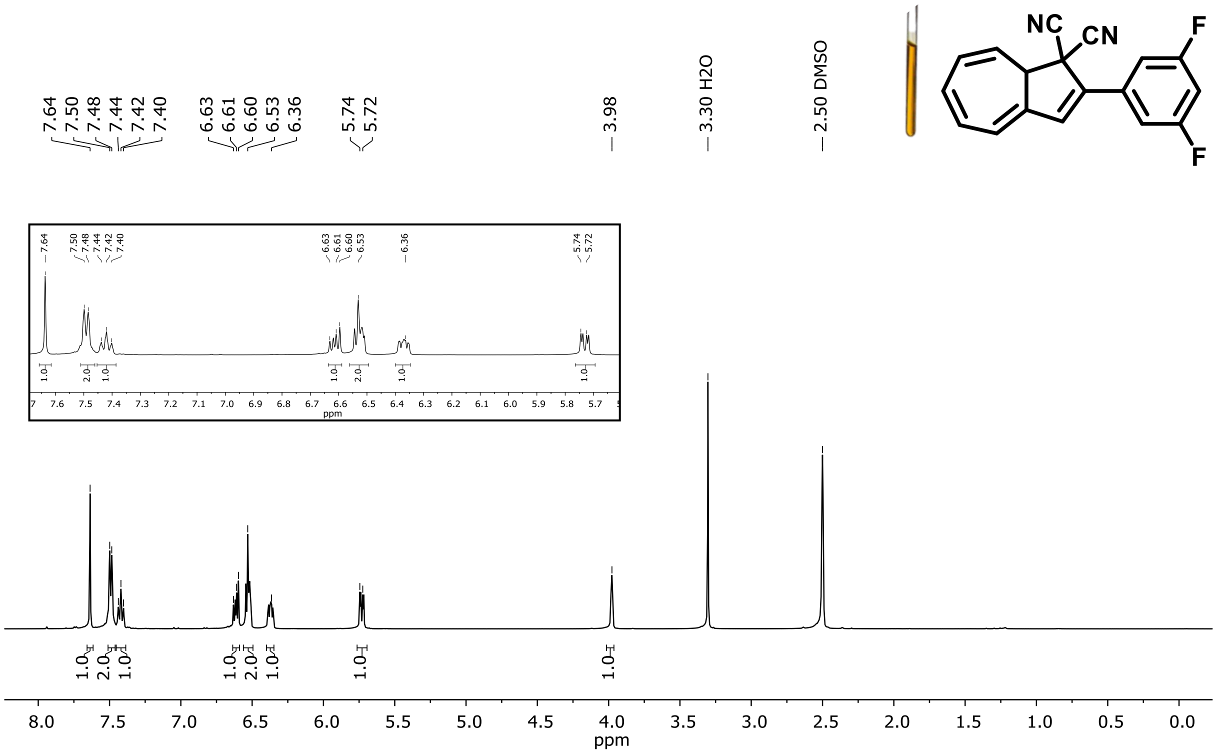

Figure S3. ^{1}H NMR of DHA-F_2 in DMSO

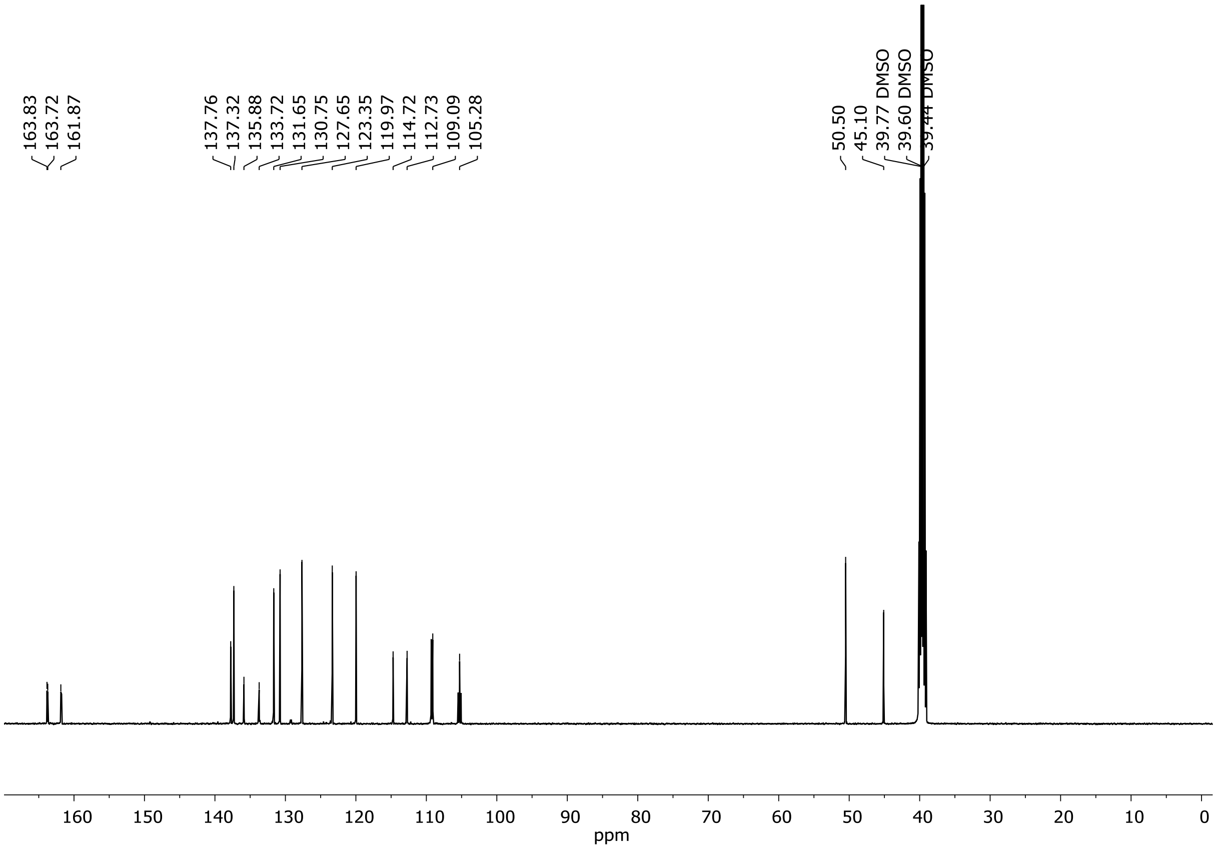

Figure S4. ^{13}C NMR of DHA-F_2 in DMSO

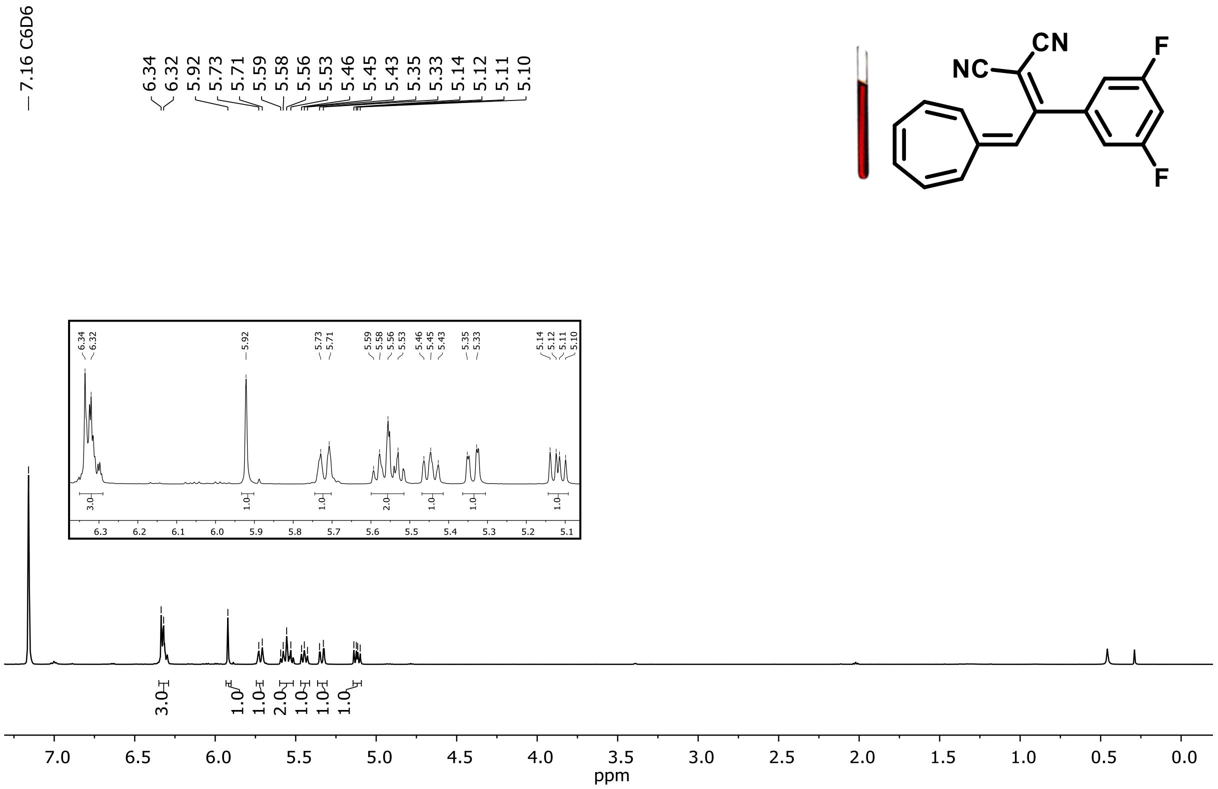

Figure S5. ^{1}H NMR of *s-cis* VHF-F_2 in C_6D_6

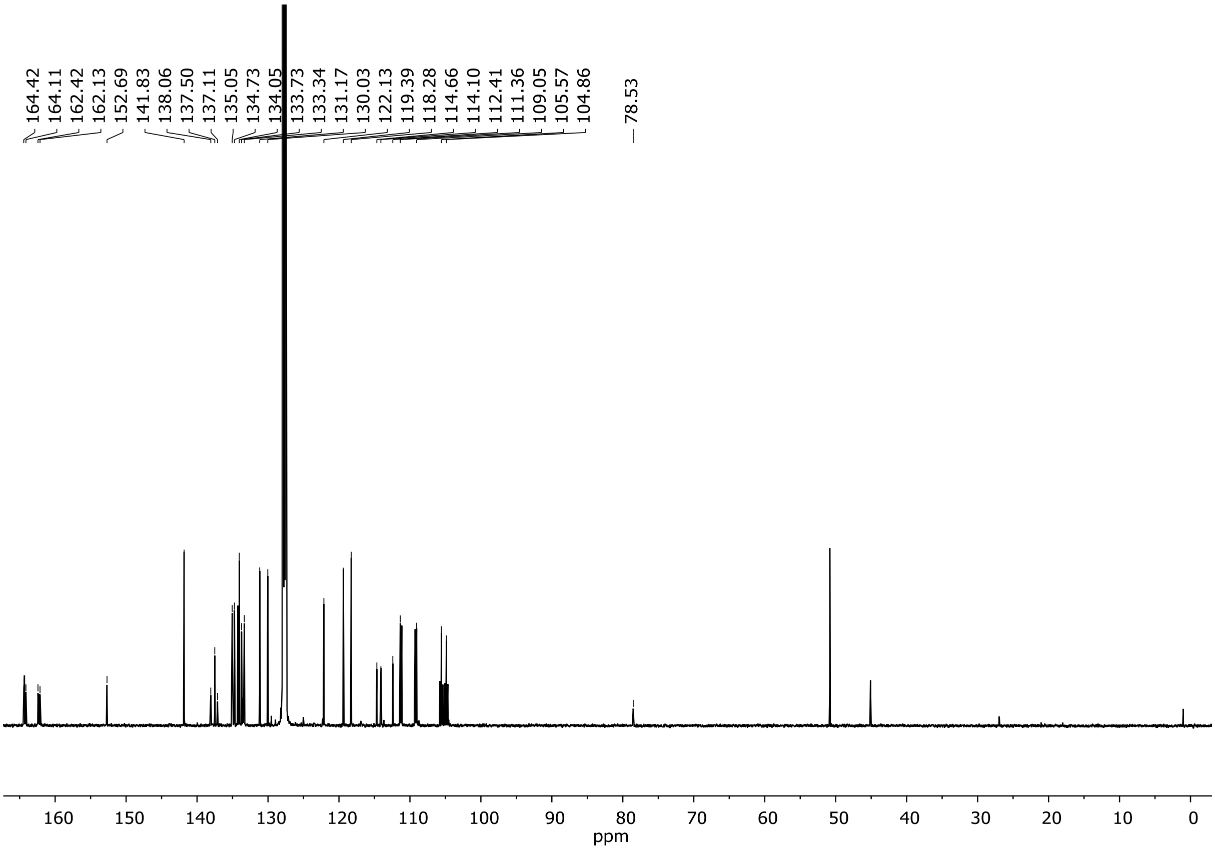

Figure S6. ^{13}C NMR of s-*cis* VHF-F_2 in C_6D_6

Thermal back reaction of VHF-F_2 monitored by ^{1}H NMR

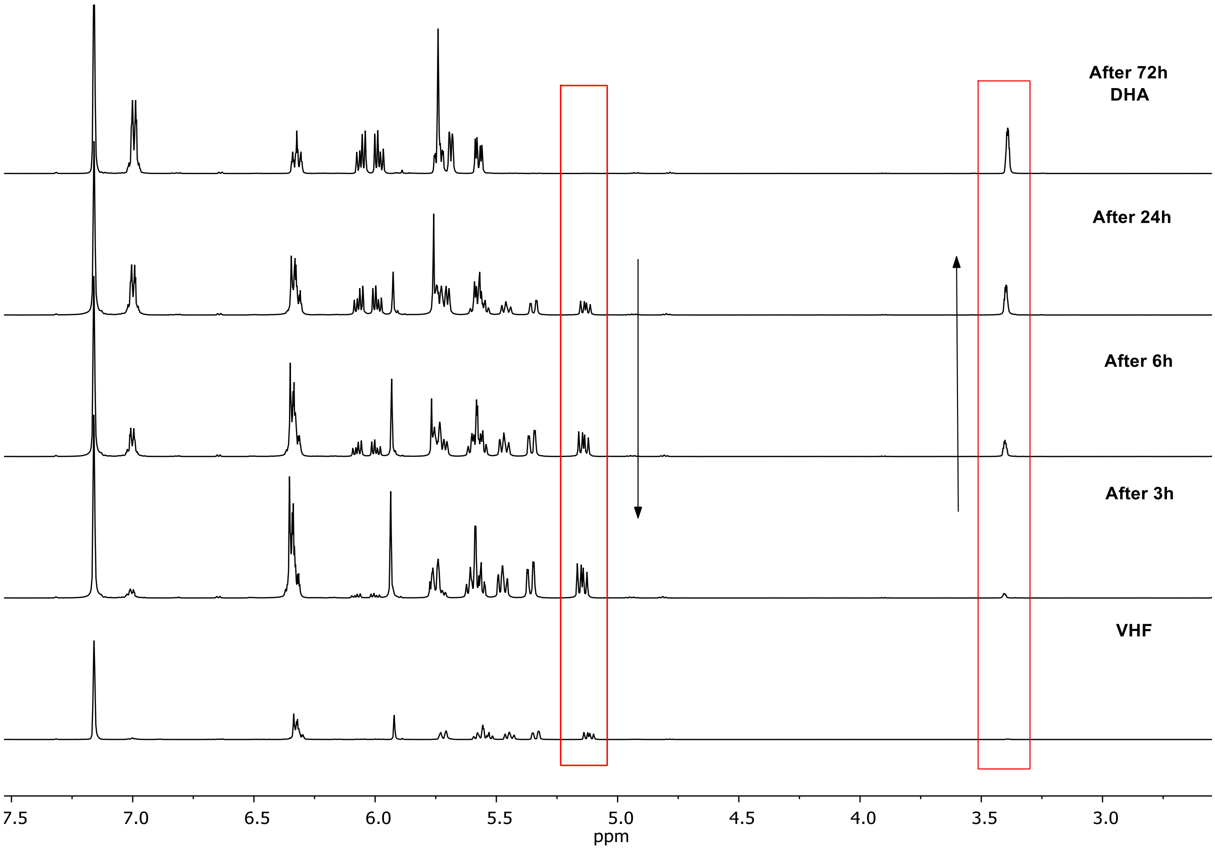

Figure S7. Stacked ^{1}H NMR spectra of the thermal back reaction from VHF-F_2 to DHA-F_2 acquired at different time interval in C_6D_6 at room temperature.

Thermal conversion of VHF-F_2 to DHA-F_2 was monitored at room temperature by acquiring ^{1}H NMR spectra every couple of hours. Figure S7 shows the decay of VHF-F_2 signals. For instance the multiplet at a chemical shift δ = 5.14-5.10 ppm belongs to VHF-F_2 and is no longer visible after 72h, while the signature signal of DHA-F_2 at δ = 3.38 ppm appears after 3h and continues to emerge over time. The complete thermally induced ring closure of VHF-F_2 at room temperature occur after 72h leading to pure DHA-F_2.

IR spectroscopy

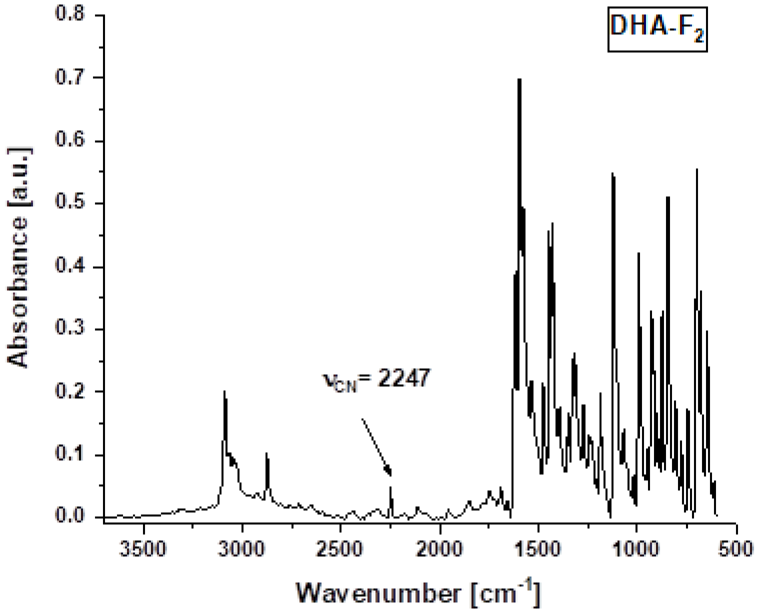

Figure S8. IR spectrum of DHA-F_2.

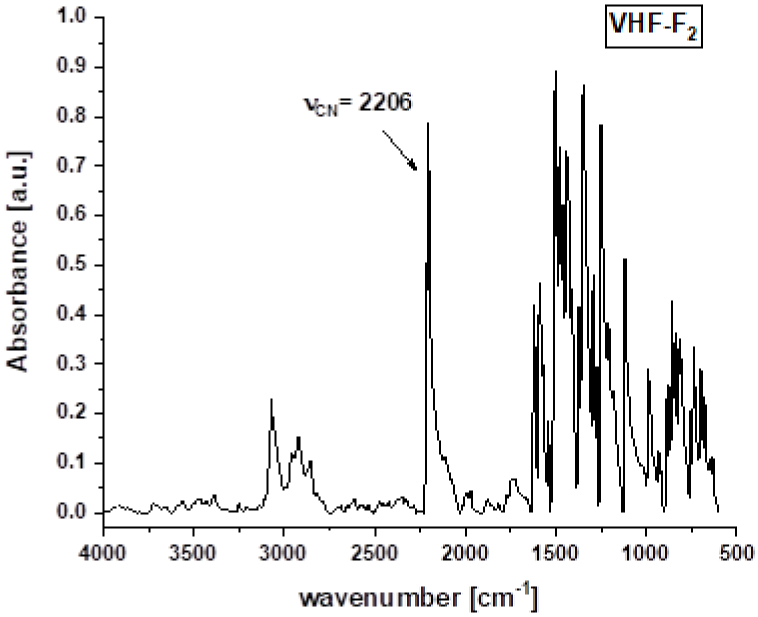

Figure S9. IR spectrum of VHF-F_2.

TGA-FTIR Data

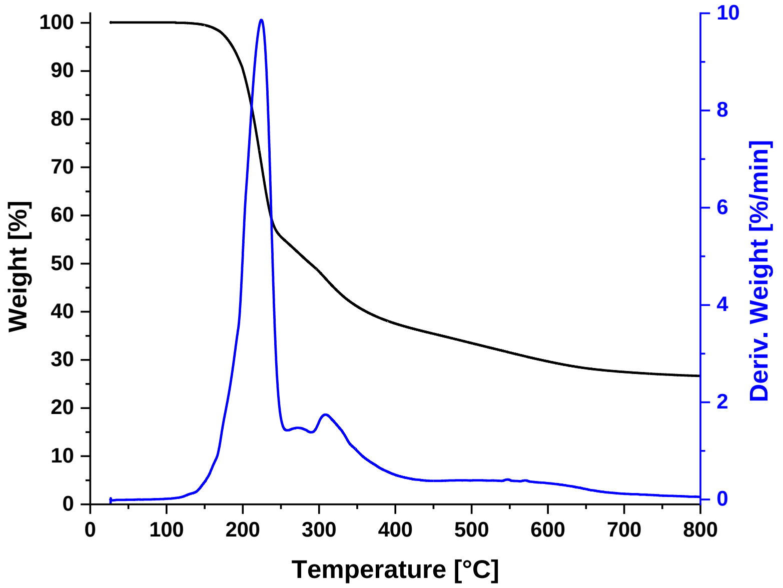

Figure S10. Weight loss of DHA-F_2 by increasing the temperature.

TGA experiments were performed. As can be seen in Figure S10, DHA-F_2 decomposition starts at temperature above 180°C (14% weight loss). The evolved gases collected from the degradation of DHA were detected by FTIR and the results are shown in The spectrum taken at 250 °C indicates the cleavage of -NC group and the formation of hydrogen cyanid, C-H stretching at $\nu = 3386\ cm^{-1}$ and H-C-N bending $\nu = 946\ cm^{-1}$. At temperatures above 250 °C, the FTIR spectra show several thermal decomposition products (e.g. aromatic compounds).

UV-Vis Spectra and switching studies

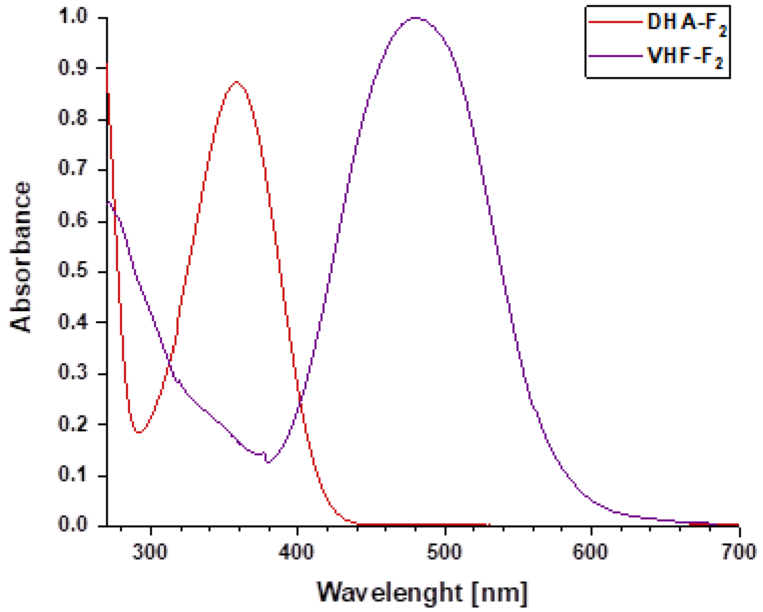

Figure S11. Absorption spectra of DHA-F_2 and VHF-F_2 in MeCN at 25°C

Switching Studies of DHA-F_2/VHF-F_2

1.6 mg of photochromic DHA-F_2 is dissolved in 25 mL acetonitrile (MeCN) and stored in the dark. 300 µL of this stock solution is placed in a quartz cuvette and the sample further diluted with 2 mL MeCN. The sample was irradiated outside of the spectrometer, before the measurement using a UV lamp (500 µW/cm² in 10 cm) equipped with a monochromator selecting 365 nm. Spectra were acquired after 2, 7, 10, 13, 17, 22, 30, 40, 60,70, 85, 100, 125,150, 175 and 200 s. By increasing the irradiation time, absorption of DHA (λ_{max}= 358nm) decrease steadily giving rise to a new absorption maximum (VHF-F_2).

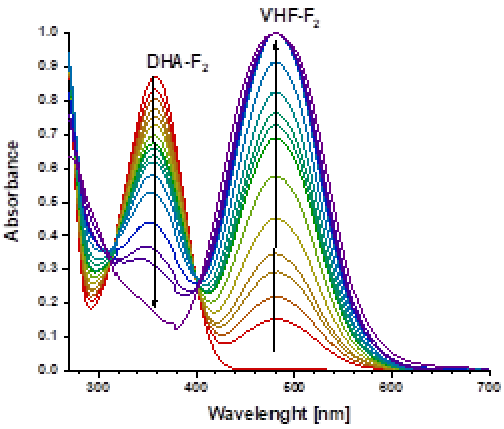

Figure S12. Conversion of DHA-F_2 to VHF-F_2 by UV irradiation over time

Calculated dipole moment in gas phase

Structure (derivative)	$\mu_{(DHA)}$ [D]	$\mu_{(VHF)}$ [D]
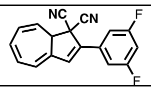	5.40	8.23
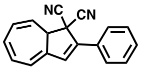	5.59	7.80
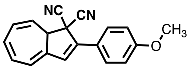	3.39	6.87
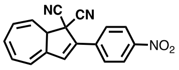	8.00	9.68

Table S1. Calculated dipole moment in Debye (D) of DHA derivatives and corresponding s-*trans*-VHF in the gas phase.

Calculated dipole moment of the observed molecules

	**Structure**	**D_{xy}**	**D_z**	**D**
Gas phase (On-surface geometry*)	s-*cis*-VHF-F_2 (**1**)	7.86	3.85	8.75
	Monocyano-DHA-F_2 (**2**)	6.5	0.01	5.55
	DHA-F_2 (**3**)	5.4	1.13	5.55

Table S1. Calculated dipole moment in Debye (D) of the three observed molecules. Note that the results are based on the calculations of molecules in gas phase with on-surface geometries.

Additional overview STM image

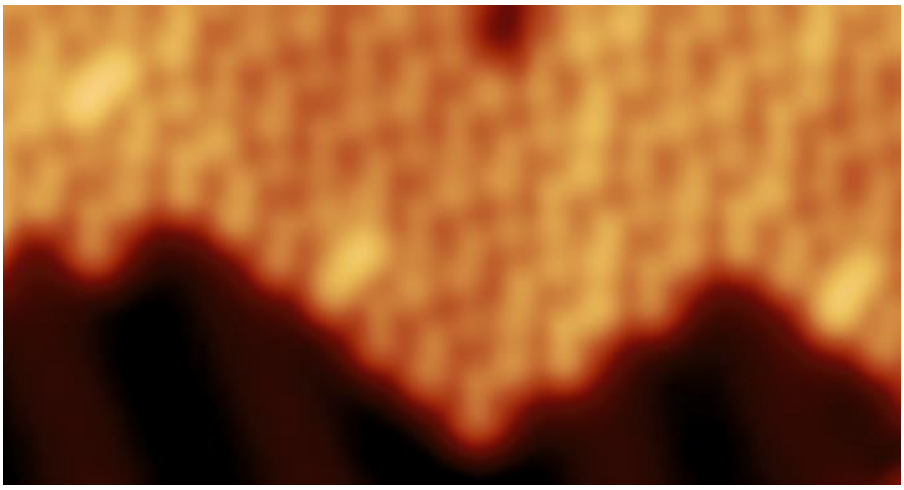

Figure S13. Additional overview STM topography of a molecular island mainly consisting the elbow-shaped molecule **1**. STM image (size: 15 nm x 8 nm) was obtained under the conditions of $V = 0.5$ V and $I = 7.7$ pA.

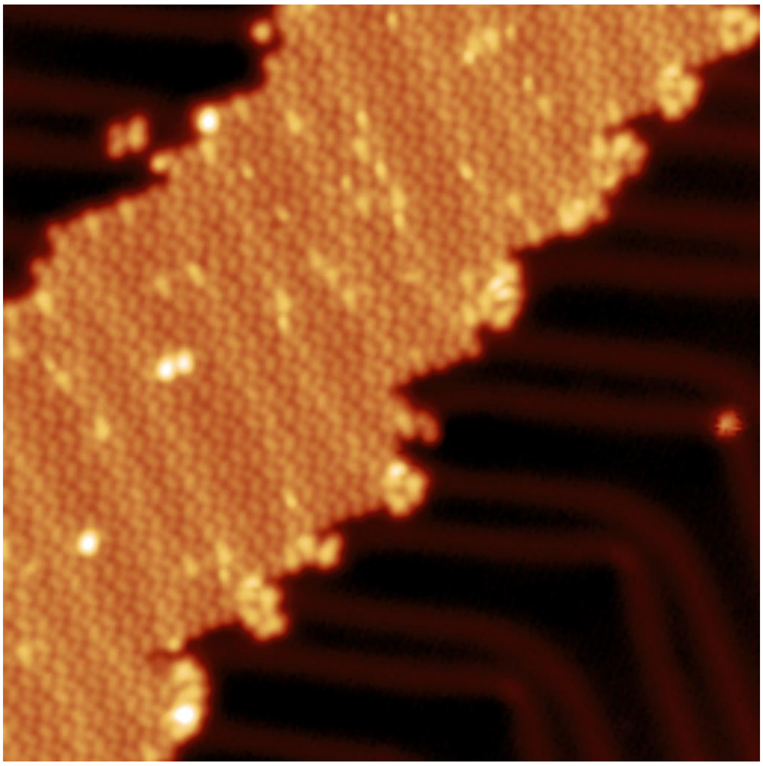

Figure S14. Additional overview STM topography of a molecular island mainly consisting the elbow-shaped molecule **1**. A few molecules **2** are visible at the island edges. STM image (size: 40 nm x 40 nm) was obtained under the conditions of $V = 0.5$ V and $I = 7.7$ pA. The image was used to estimate the relative amount of molecules **1**, **2**, and **3** on the surface.

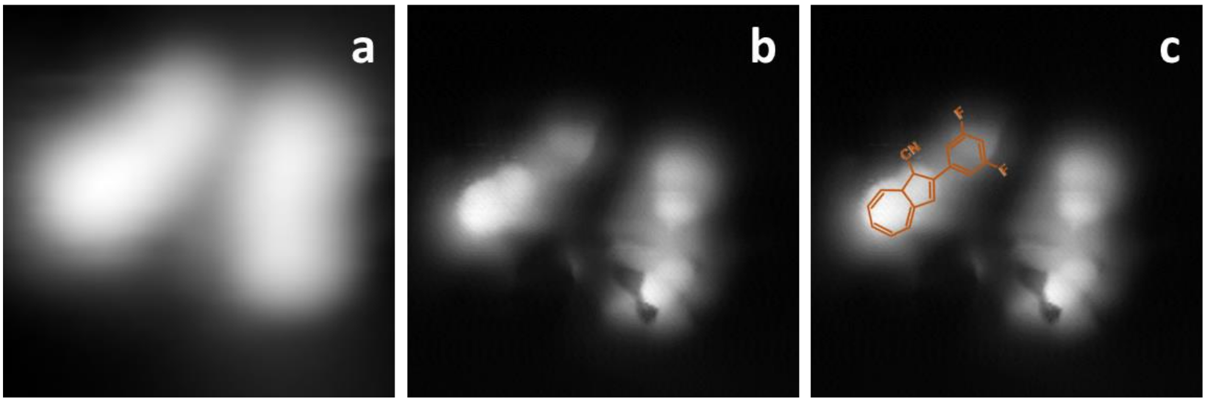

Figure S15. High-resolved STM images of molecule **2**. (a) Constant current STM topography (size: 3 nm x 3 nm, V = 0.5 V, I = 7.7 pA). (b – c) Constant-height high resolution (CO-functionalized tip) STM image (size: 3 nm× 3 nm, V = 10 mV) and the corresponding superimposed structure of molecule **2**. On the right molecule, an STM artifact is visible due to an unexpected movement of **2** (right) during scanning.

Isolation of single molecule 1

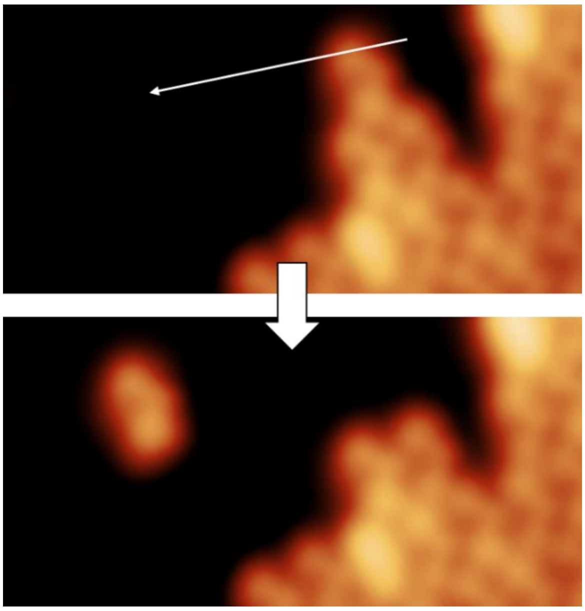

Figure S16. Isolation of single molecule **1** from the edge of the molecular island. (Top) White arrow in the manipulation sequence indicate trajectories of lateral manipulations in constant current mode (V = 10 mV; I = 8 pA). (Bottom) The subsequent image shows that the molecule has been successfully isolated from the island, following the trajectory of the tip. STM images (10 nm x 5 nm) were obtained under the conditions of V = 0.5 V and I = 7.7 pA.

Additional data of switching molecule 1 to 2

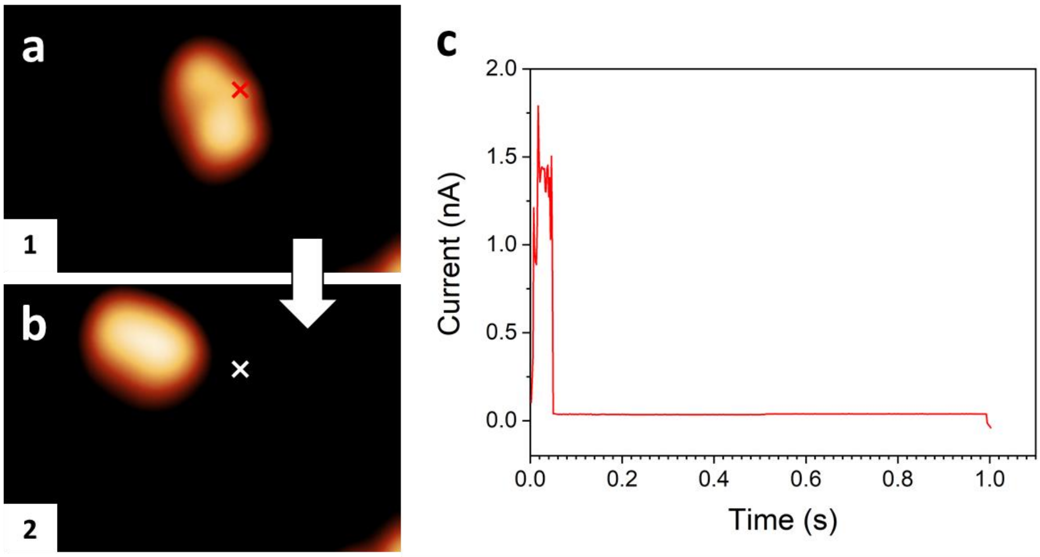

Figure S17. STM voltage pulse induced switching of a single molecule shown in Figure 4b - c and the corresponding current signal. By applying a 2.5 V voltage pulse above **1** in constant height mode (marked), a sudden drop of current can be recorded. However, such sudden drop of the current signal records simultaneously both the conversion and movement events. All STM images (6 nm × 4 nm) were obtained under the conditions of V = 0.5 V and I = 7.7 pA.

Tip-induced movement sequence before and after conversion

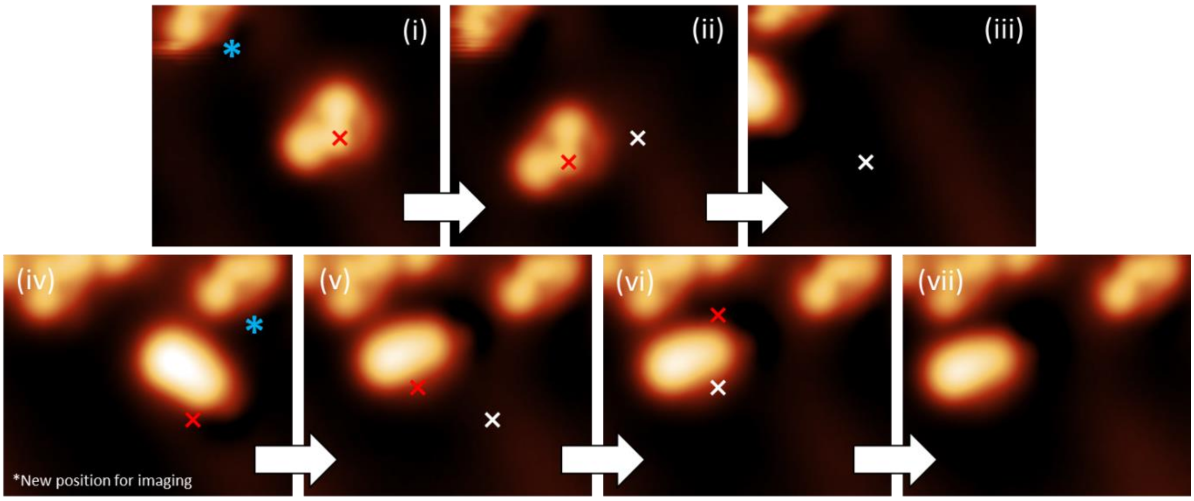

Figure S18. STM voltage pulse induced switching and movement. (i) Subsequent STM image after isolated a single molecule **1** from the edge of a molecular island. The blue star represents a reference point. (i) – (ii) Two individual voltage pulses (red mark; V = 2 V, I = 1.2 nA) are applied above this molecule, where lateral displacements can be observed. The red and white marks indicate the position of the tip before and after applying the voltage pulses, respectively. (iii) After a pulse at (ii), a subsequent image was obtained, a change of appearance can be seen, however, the molecule is out of the scanning range. (iv) After moving the STM tip to the left

in order to capture the target molecule, molecule **2** is observed, meaning a successful switching from **1** to **2**. The blue star represents the same reference point as (i). (v) – (vii) Further voltage pulses at different parameters (V = 1 – 1.2 V, 100 – 200 pA) were applied. No controllable movement of molecule **2** can be seen. All STM images (6 nm x 5 nm) were obtained under the conditions of V = 0.5 V and I = 7.7 pA.

Additional data of moving molecule 1

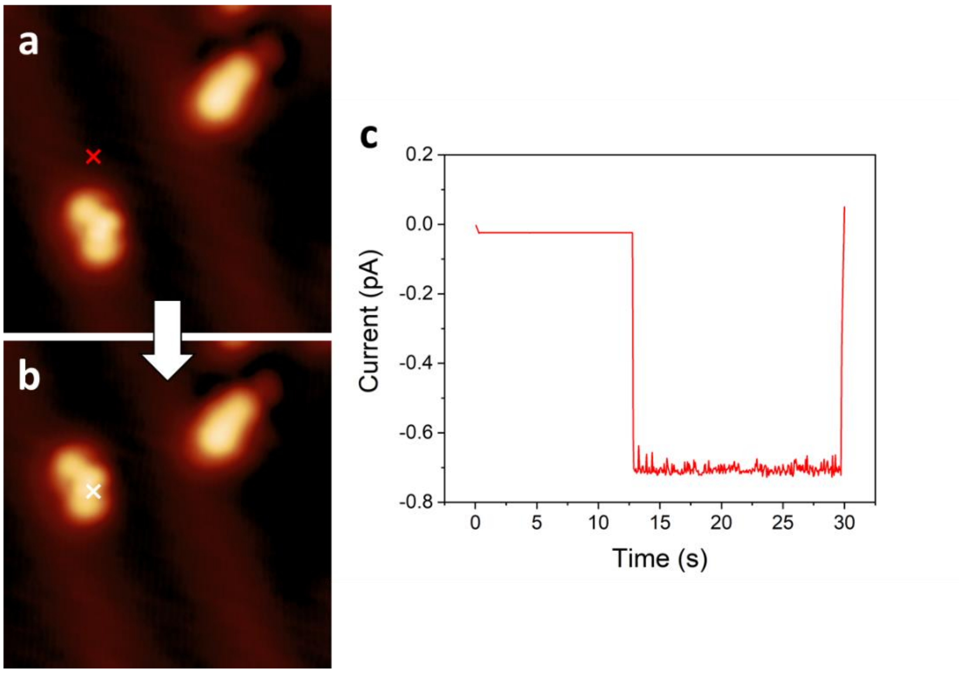

Figure S19. Exemplary STM voltage pulse induced movement. (a) By applying a -1.8 V voltage pulse near to **1** in constant height mode, a sudden increase of current can be recorded as shown in (c). (b) Subsequent STM image shows a lateral displacement of **1** attracted by the tip. The red and white marks indicate the position of the tip before and after applying the voltage pulses, respectively. All STM images (6 nm × 4 nm) were obtained under the conditions of V = 0.5 V and I = 7.7 pA.

Calculations with applied electric field

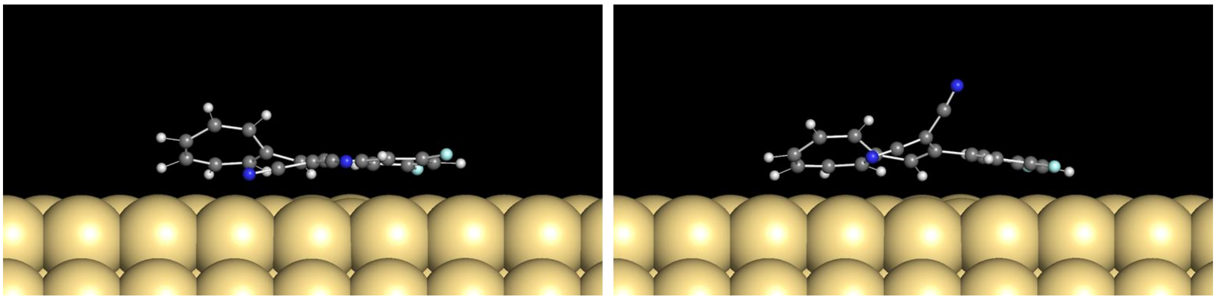

Figure S20. To understand the pulse-induced conversion from molecule **1** to **2**, we calculated the adsorption of s-*cis*-VHF-F_2 applying an external electric field perpendicularly to the surface. The left panel shows the adsorption of s-*cis*-VHF-F_2 on Au(111) without electric field. If one applies a positive field (we call it positive if the substrate has positive potential due to the tip) the molecule is pulled up, and at an electric field of 1 V/ Å (right panel) the force is definitely applied to one of the CN groups, with the consequent breaking of the C-C bond, the elimination of HCN, and closing of the ring. For the opposite direction of the field, the effect of the external electric field is much weaker in agreement with the experiment.
